# The disease burden in patients with respiratory allergies induced by house dust mites: a year-long observational survey in three European countries

**DOI:** 10.1186/s13601-020-00331-0

**Published:** 2020-07-01

**Authors:** Pascal Demoly, Andrea Matucci, Oliviero Rossi, Carmen Vidal

**Affiliations:** 1 Allergy Division, Pulmonary Department, Hôpital Arnaud de Villeneuve, University Hospital of Montpellier, 371 Avenue du Doyen Gaston Giraud, 34090 Montpellier, France; 2 Sorbonne Université, UMR-S, 1136 INSERM, IPLESP, Equipe EPAR, Paris, France; 3 Department of Internal Medicine, Section of Immunoallergology and Respiratory Diseases, University of Florence, Florence, Italy; 4 Allergy Service, Complejo Hospitalario Universitario de Santiago, Santiago de Compostela, Spain

**Keywords:** Respiratory allergy, House dust mite, Long-term observational survey, Burden of disease

## Abstract

**Background:**

House dust mite (HDM) allergens constitute the most frequent cause of persistent allergic rhinitis and asthma. The symptoms vary throughout the year but typically peak in spring, autumn and (to a lesser extent) mid-winter.

**Methods:**

We performed a 13-month, observational, multicentre survey of adult patients with a self-reported history of moderate-to-severe, poorly controlled, physician-diagnosed HDM respiratory allergy in three European countries (France, Italy and Spain). After screening and inclusion, 28 detailed, fortnightly telephone interviews were used to gather extensive data on the participants’ symptom prevalence and intensity, medical consultations, disease burden and medication use from late May 2012 to early July 2013. This report focuses on the disease burden.

**Results:**

Of the 22,995 screened participants, 313 met the inclusion criteria and completed the post-inclusion questionnaire (*n* = 114 in Italy, 92 in France and 107 in Spain). The median time since the first symptoms of HDM allergy was ≥ 13 years in each country. A relevant minority of the participants suffered from symptoms of HDM allergy every day or almost every day of the year (14% in Italy, 46% in France and 37% in Spain). According to the fortnightly telephone interviews, the most frequently impacted disease burden variables were sleep, daytime tiredness and irritability, with the highest values in spring 2012, autumn 2012 and spring 2013 (mirroring symptom intensities). Professional activities were more affected than social activities. The burden data were heterogeneous: around a quarter of participants were strongly or very strongly affected but most of the remaining participants were only rarely bothered or not bothered.

**Conclusions:**

In a 13-month, fortnightly survey of patients in France, Italy and Spain with a self-reported history of moderate-to-severe, poorly controlled, HDM-induced allergic rhinitis and asthma, we found that a relevant minority of participants regularly reported a severe or very severe impact of their allergy on tiredness, sleep and professional activities (including time off work). The disease burden peaked in autumn and late spring.

## Background

House dust mite (HDM) allergens constitute the prime cause of respiratory allergies (i.e., allergic rhinitis (AR) and allergic asthma) [1, 2]. These conditions affect more than 500 million people worldwide, albeit with significant geographical variations in exposure and seasonality [3, 4]. Although the symptoms of HDM-induced respiratory allergy are rarely absent, their intensity varies over time as the indoor HDM populations and allergen levels fall or rise as a function of changes in the weather or the domestic environment [5, 6]. Patients consulting an allergist for HDM allergy tend to have moderate-to-severe disease profiles, with high levels of polysensitization and (potentially) polyallergy. In a French, retrospective survey of 1289 HDM-allergic patients receiving allergen immunotherapy, 62.5% of the patients were polysensitized and 50% suffered from asthma [7]. Similarly, in a prospective, observational, multicentre, cross-sectional study of 1212 HDM-allergic patients conducted in France between 2013 and 2014, 57.5% of the participants were polysensitized and 42% had asthma [8]. The frequent presence of asthma was also confirmed by a study of 1212 HDM-allergic patients (median age: 22 years; 52% women) in France (one of the study countries in the present research) selected for allergen immunotherapy (AIT); 42% suffered from asthma [8]. Likewise, a study of HDM-allergic, AIT-eligible patients in Italy (also a study country in the present research) found an asthma prevalence of 41% [9].

The complexity and severity of HDM allergy is illustrated by the fact that whereas patients with grass-pollen allergy tend to consult a general practitioner, HDM-allergic patients tend to consult a specialist physician [10].

Very few detailed studies have addressed the complexity of the disease profile in HDM respiratory allergy. In a longitudinal, single-centre study in Sydney (Australia), changes in the intensity of HDM-induced symptoms were recorded over a 1-year period [11]. Thirty-seven patients completed the study. The researchers observed pathologically high nasal symptom scores for 65% of the 12-month study period. The nasal symptom scores were predictive of the use of nasal medications.

In view of the importance of this topic and the limitations of a previous study in Australia (a small number of participants, a single-centre design and a small number of outcomes), we performed an observational, multinational, multicentre survey of adult patients with a history of moderate-to-severe, poorly controlled, physician-diagnosed HDM respiratory allergy who had not received allergen immunotherapy [12]. The study took place in three Western European countries: France, Italy and Spain. Fortnightly telephone interviews were used to gather data on symptom prevalence and intensity, medical consultations, disease burden, quality of life (QoL) and medication use over a 13-month period of ‘real life’. Our results concerning the study population’s baseline characteristics and the subsequent changes over time in the prevalence of nasal and ocular symptoms, the worsening of symptoms and physician consultations were published recently [12].

Regulatory bodies and patient associations are placing ever-greater emphasis on patient-reported outcomes such as QoL. The symptoms of HDM-induced allergy are known to have a significant negative impact on QoL [13]. Here, we present data on the burden of disease at baseline and over the subsequent 13-month survey period.

## Methods

**Fig. 1:**
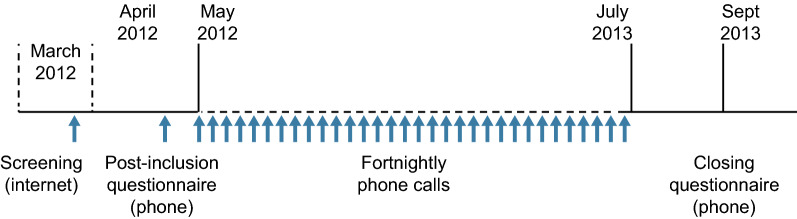
Study timeline

The design and procedures of this observational, Internet- and telephone-based survey have been described in detail elsewhere (Fig. 1) [12]. In each country, members of nationwide patient panels (previously constituted by the study’s contract research organisation STETHOS (Sèvres, France)) were invited to participate. In these three countries, we were informed that specific ethical and regulatory approval from independent ethics committees or health authorities was not required for a non-interventional, anonymous survey. The contract research organisation complied with national legislation (notably with declarations to the national data protection committees and national medical associations) and industry guidelines and codes of good practice. Each panel member had a unique identifier. The anonymised format of the study database prevented the direct, nominative identification of panel members. The survey participants had provided their prior general consent to exploitation of the anonymised data. Participants screened themselves for eligibility with a short Internet questionnaire and had to confirm that they had been diagnosed with HDM allergy by a physician. Participants received modest remuneration for their participation.

The study’s inclusion criteria were as follows: age 18 years or over; moderate-to-severe symptoms of HDM allergy; at least three of the following symptoms: blocked nose, runny nose, itchy nose, difficulty breathing, cough, wheezing, sneezing, chest tightening, itchy eyes and tearing; physician-diagnosed HDM allergy (and potentially other allergies); a positive skin prick test or a specific serum immunoglobulin E assay for HDM allergens; more severe allergic symptoms in September, October, November or December; no previous or current allergy immunotherapy; use of at least one antihistamine or corticosteroid medication; symptoms not sufficiently controlled by current medication; a moderate to very strong impact of HDM allergy on QoL.

All survey questionnaires (the nine-question Internet-based screening questionnaire, the 28-question telephone-based post-inclusion questionnaire, the 10-question fortnightly telephone-based interview and the 24-question telephone-based final questionnaire) were drafted in English, translated into local languages (Italian, French and Spanish) and then translated back into English for validation [14]. Most of the study data presented below were generated via the fortnightly telephone interview, which was administered from late May 2012 to early July 2013.

In the fortnightly telephone interview (but not in the post-inclusion and final questionnaires), disease control was assessed with the validated Allergic Rhinitis Control Test (ARCT) [15]. The ARCT is based on five items (scored from 1 ‘permanently’ to 5 ‘never’) assessing the patient’s AR over the previous fortnight, which yields a score from 5 (the lowest possible degree of control) to 25 (the highest possible degree of control). In the clinical validation of the ARCT, disease control was defined as a score of 21 or more [15].

A descriptive analysis of the survey data was performed using SPSS software (version 15.0.1, IBM Corporation, Armonk, USA). Quantitative parameters are expressed as the mean, median and range, and qualitative parameters are expressed as the number and the percentage of the corresponding survey population or subpopulation.

## Results

### The impact of HDM allergy at baseline, according to the post-inclusion questionnaire

A total of 22,995 individuals completed the Internet screening questionnaire. Of these, 339 (1.5%) met all the inclusion criteria and 313 (*n* = 114 in Italy, 92 in France and 107 in Spain) were included in the study and completed the post-inclusion questionnaire. Within each country, the included participants were evenly distributed in terms of their place of residence (coast vs. inland, north vs. south, etc.). The baseline characteristics of the study population have been published in our initial report on the study but are summarised again here for convenience (Additional file 1: Table S1) [12]. The study population was predominantly female (67% women), and the mean age was 37.2 years (which is similar to the value in the Australian study) [11, 12]. The median time since the onset of HDM allergy symptoms was 13 years or more in each country. The participants tended to consult a general practitioner (rather than a specialist physician) when their allergic symptoms worsened (according to 53% of the participants in Italy, 51% in France and 47% in Spain), which could be because of the easier accessibility to the former with respect to the specialist. According to the more detailed telephone-based post-inclusion questionnaire (which notably included an opportunity to ask the person conducting the interview to clarify the meaning of questions, if required), the vast majority of participants were taking more than one medication for their allergy (80%, 85% and 80% in Italy, France and Spain, respectively). Between 4% and 11% of the participants (depending on the country) reported total disease control (although the level of control was not defined with a validated instrument in this questionnaire) and between 43% and 53% reported good disease control (Additional file 1: Table S1). A relevant minority of the participants suffered from symptoms of HDM allergy every day or almost every day of the year (14% in Italy, 46% in France and 37% in Spain). According to the self-reports in the post-inclusion questionnaire, the study participants in all three countries noticed spring and autumn peaks in their symptoms (thus confirming one of the inclusion criteria), as is often observed in the literature [6, 16]. Most participants were taking more than one medication for their allergy (80%, 85% and 80% in Italy, France and Spain, respectively).

**Fig. 2:**
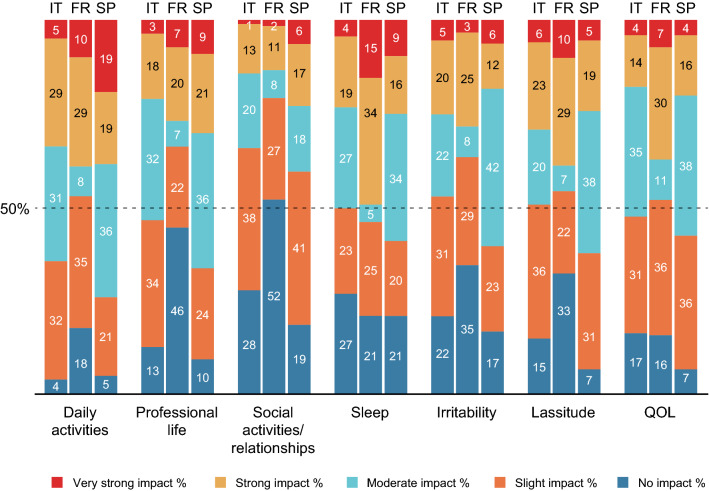
Impact of symptoms on seven types of life activity at baseline (as self-reported by participants in the post-inclusion questionnaire)

In the post-inclusion questionnaire, the participants were asked to rate the current impact of the symptoms of HDM allergy on their daily activities, professional activities, social life, sleep, irritability, lassitude and QoL (Fig. 2). Overall, daily activities and sleep were most strongly affected. There were some marked intercountry differences: the proportion of participants reporting a strong or a very strong impact of HDM allergy on daily activities was 34% in Italy, 39% in France and 38% in Spain. With regard to sleep, the proportion of participants reporting a strong or a very strong impact was notably greater in France (49%) than in Italy (23%) or Spain (25%). Similarly, the proportion of participants reporting a strong or a very strong impact on QoL was markedly higher in France (37%) than in Italy (28%) or Spain (20%). Social activities and relationships were least affected, with a strong or a very strong impact reported by 14% of the participants in Italy, 13% in France and 23% in Spain.

### Changes in the impact of HDM allergy over a year-long period, as reported by participants in the fortnightly telephone interviews

Every 2 weeks, the study participants underwent a detailed telephone interview on the intensity of nasal, ocular and respiratory symptoms and the latter’s impact on a number of daily activities and QoL parameters. The time trends for the nasal, ocular and respiratory symptoms were similar: the fortnightly prevalence fell during the summer of 2012, rose in the autumn (peaking in early October), fell over the winter, peaked again in the spring of 2013, and then fell over the summer, as previously reported [12].

To rule out interference by other sensitizations in the seasonality observed for the symptoms, ‘HDM-only’ patients (i.e. the 85 patients in whom HDM allergy was the only self-reported allergy; *n* = 44 in Italy, 19 in France and 22 in Spain) were analysed separately. As seen in the overall study population, the symptom scores in in the ‘HDM-only’ population showed peaks in spring and autumn and troughs in summer and winter.

**Fig. 3:**
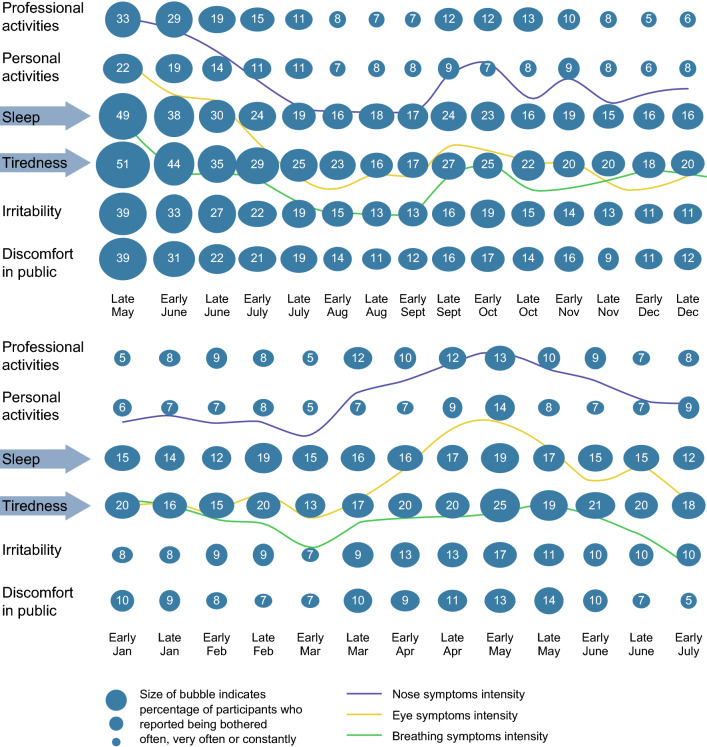
Fortnightly telephone interviews of study participants: the change over time in the impact of allergy on six types of life activity, from late May to December 2012 (top panel) and from January to July 2013 (bottom panel)

The self-reported impact on six disease burden indicators mirrored the time trends for symptom intensity (Fig. 3 for all participants and Additional file 2: Figures S1 and Additional file 3: Figure S2 for ‘HDM-only’ patients). The proportion of participants who reported being bothered often, very often or constantly was highest at the start of the fortnightly follow-up period (i.e. the late spring of 2012) and then decreased over the summer. We observed a slight increase in the autumn of 2012 and then again in the spring of 2013.

The most frequently affected activities were sleep, daytime tiredness and (to a lesser extent) irritability (Fig. 3 for all participants and Additional file 2: Figures S1 and Additional file 3: Figure S2 for ‘HDM-only’ patients). According to the reports in the post-inclusion questionnaire, personal/social activities were least frequently affected by HDM allergy. In contrast, between one-half and two-thirds of the participants (depending on the parameter) were never bothered or only sometimes bothered by their symptoms. This reveals great heterogeneity in the disease profiles.

**Fig. 4:**
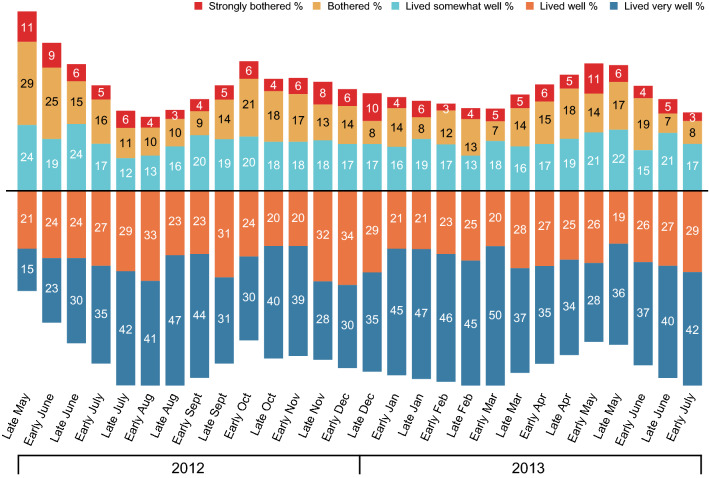
Fortnightly telephone interviews of study participants: overall feeling about the bothersomeness of HDM allergy

The participants’ overall feelings about the burden of disease also mirrored the changes in symptom intensity, with a relatively high level of impairment in late spring 2012, a rapid decrease over the summer, a peak in the autumn and a second peak in the spring of the following year (Fig. 4). Again, the self-reported data were heterogeneous: except at the very start of the survey, more than half of the participants were not bothered or were only rarely bothered by their symptoms. The time trends for the 85 HDM-only patients (data not shown) were again similar to those seen in the overall study population, with peaks in bothersomeness in autumn and spring. Somewhat surprisingly, a minority (15%) of the participants indicated that they ‘lived very well’ in late May 2012, despite the fact that (i) insufficient disease control with the current medication and (ii) a moderate to very strong impact of HDM allergy on QoL were inclusion criteria. The proportion who ‘lived very well’ had risen to 47% by late August 2012.

**Fig. 5:**
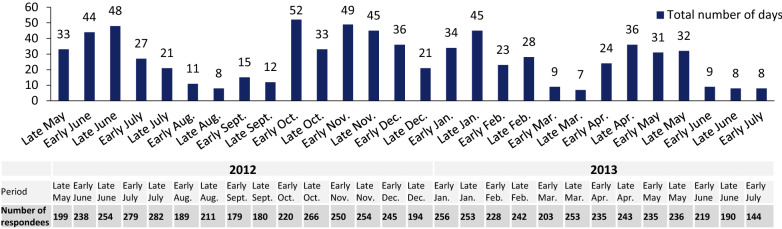
Fortnightly telephone interviews of study participants: total number of days off work in each fortnightly period (*n* = 313 participants)

Participants consistently reported being bothered more in their professional activities than in their personal activities. The fortnightly telephone interview also assessed a measure of the disease burden in professional life: the total (pooled) number of days off work per fortnight in the study population (Fig. 5). The change over time in this parameter mirrored the above-described changes in symptom intensity and disease burden, with a decrease over the summer of 2012, a peak in the autumn and a second peak in spring 2013. Very similar changes over time were observed when considering the percentage of patients who reported having missed at least one day of work in a given fortnightly period (Additional file 3: Figure S2). Overall, fewer than 10% of the participants reported having missed at least 1 day of work per fortnight. The proportion of patients missing at least 1 day of work in a given fortnightly period appeared to be lower in France than in Spain and Italy.

**Fig. 6:**
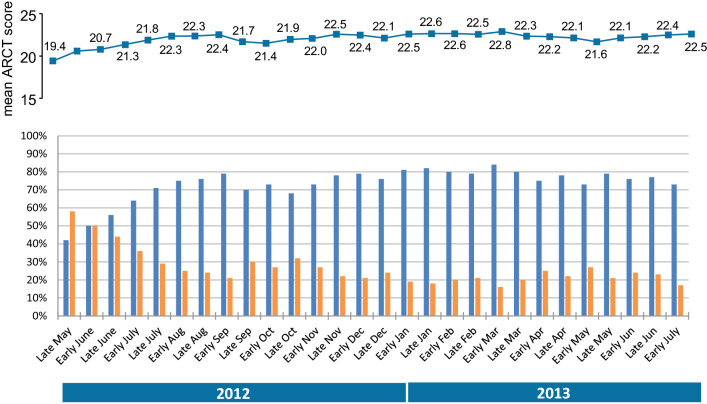
Fortnightly telephone interviews of study participants: mean ARCT score (top panel) and percentage of patients with controlled/uncontrolled disease (bottom panel)

With regard to disease control, the mean ARCT score appeared to be relatively stable over the year-long observation period (Fig. 6), with values of between 19 and 23 out of 25. However, given that disease control corresponds to a score of 21 or more, the proportion of patients with self-reported, controlled disease varied considerably (from 42% to 83%). Conversely, the proportion of patients with uncontrolled disease (score: from 5 to 20) ranged from 58 to 17% (Fig. 6). Overall, the level of disease control appeared to increase slightly over the study period. When asked to rate the level of disease control on a 0 (low) to 10 (high) numerical scale, the mean scores in Italy, France and Spain were, respectively, 6.0, 7.0 and 6.6.

For the study population as a whole and for the ‘HDM-only’ participants, the frequency of medication use remained relatively constant over the study period (Tables 1 and 2 for ‘HDM-only’ participants in 2012 and 2013, respectively). Unsurprisingly, antihistamines were most commonly administered medications (by around 40% of participants in any fortnightly period), followed by nasal corticoids (administered by around 15% of participants). All other types and classes of drug were infrequently used (less than 10% of participants).

**Table 1:** Proportions of “HDM-only” patients using the indicated medications during each questionnaire period (2012)

	2012														
	Late May	Early June	Late June	Early July	Late July	Early Aug.	Late Aug.	Early Sept.	Late Sept.	Early Oct.	Late Oct.	Early Nov.	Late Nov.	Early Dec.	Late Dec.
Antihistamines	41%	45%	42%	42%	43%	47%	47%	41%	45%	38%	36%	41%	44%	40%	46%
Nasal corticoids	14%	14%	15%	13%	16%	9%	16%	20%	12%	10%	14%	17%	13%	16%	13%
Inhaled corticoids	1%	1%	1%	–	1%	–	–	–	–	5%	–	1%	3%	–	2%
Bronchodilators	5%	1%	6%	2%	5%	12%	5%	7%	6%	5%	5%	4%	7%	5%	–
Inhaled corticoid + bronchodilator	1%	3%	2%	5%	6%	5%	7%	12%	3%	6%	3%	1%	4%	5%	4%
Other corticoids	4%	–	4%	7%	6%	5%	2%	–	–	4%	2%	–	4%	2%	8%
Eye drops	3%	3%	5%	4%	3%	5%	5%	2%	9%	5%	8%	10%	7%	8%	6%
Leukotriene receptor antagonist	3%	3%	2%	4%	3%	2%	5%	2%	6%	3%	3%	1%	3%	3%	2%
Nasal decongestants	5%	3%	6%	12%	6%	2%	4%	–	3%	6%	9%	9%	3%	8%	2%
Other anti-H1	4%	4%	1%	2%	1%	5%	–	–	3%	1%	3%	3%	3%	–	–
Other nasal sprays	7%	3%	4%	1%	3%	2%	2%	7%	6%	3%	3%	1%	1%	3%	2%
Cromone	–	1%	–	–	–	–	–	–	–	–	–	–	–	–	–
Cough syrups	1%	1%	1%	–	–	2%	–	–	-+	–	–	–	1%	–	–
Pain killers	–	1%	1%	1%	–	2%	–	5%	–	3%	3%	1%	1%	3%	6%
Antimigraine drugs	1%	4%	1%	1%	–	–	–	–	–	–	–	–	–	–	–
Homeopathy	–	–	–	–	–	–	–	–	–	–	–	1%	–	–	2%
Antibiotics	3%	3%	2%	1%	1%	–	–	–	–	1%	2%	–	1%	–	–
Other	7%	5%	6%	4%	0%	7%	4%	2%	6%	10%	13%	7%	4%	6%	8%

**Table 2:** Proportions of “HDM-only” patients using the indicated medications during each questionnaire period (2013)

	2013												
	Early Jan.	Late Jan.	Early Feb.	Late Feb.	Early Mar.	Late Mar.	Early Apr.	Late Apr.	Early May	Late May	Early June	Late June	Early July
Antihistamines	42%	37%	38%	37%	38%	44%	46%	39%	43%	39%	32%	33%	42%
Nasal corticoids	12%	17%	18%	18%	17%	13%	16%	13%	10%	14%	15%	13%	6%
Inhaled corticoids	3%	2%	2%	2%	2%	2%	2%	1%	–	2%	1%	2%	3%
Bronchodilators	4%	7%	5%	6%	8%	–	2%	4%	5%	8%	7%	5%	3%
Inhaled corticoid + bronchodilator	4%	5%	6%	4%	2%	7%	2%	3%	5%	6%	6%	5%	3%
Other corticoids	4%	–	5%	2%	2%	5%	3%	5%	1%	3%	4%	–	–
Eye drops	4%	5%	2%	8%	6%	3%	5%	5%	9%	6%	6%	7%	6%
Leukotriene receptor antagonist	3%	5%	5%	2%	6%	3%	5%	5%	5%	5%	4%	4%	6%
Nasal decongestants	7%	13%	6%	8%	4%	7%	5%	8%	6%	5%	8%	5%	9%
Other anti-H1	1%	2%	–	–	2%	2%	–	1%	1%	–	–	–	–
Other nasal sprays	3%	2%	–	2%	–	–	2%	3%	5%	–	1%	4%	6%
Cromone	–	–	–	–	–	–	–	–	–	–	–	–	–
Cough syrups	–	–	2%	–	–	–	–	–	–	–	3%	–	–
Pain killers	3%	2%	3%	–	4%	2%	3%	4%	3%	5%	3%	7%	3%
Antimigraine drugs	1%	–	–	–	–	–	–	–	–	–	1%	–	–
Homeopathy	1%	–	–	–	–	–	–	–	–	2%	–	–	–
Antibiotics	–	–	–	2%	–	–	–	–	–	–	–	–	–
Other	8%	5%	16%	8%	12%	18%	13%	9%	7%	9%	7%	14%	15%

### Changes in the impact of HDM allergy over the study period, as reported in the final questionnaire

**Fig. 7:**
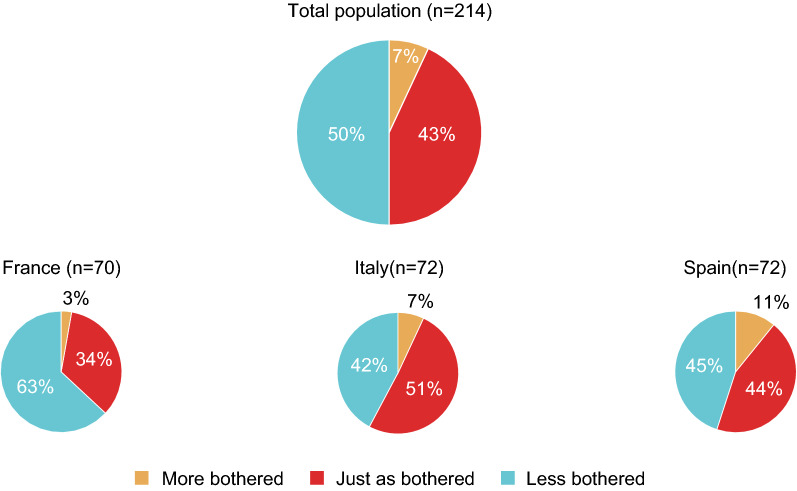
Overall change in the disease burden of HDM allergy symptoms during the study period (according to the final questionnaire) for the study population as a whole and in each country

A total of 214 participants completed the final questionnaire (*n* = 72 in Italy, 70 in France and 72 in Spain). The main reasons for not completing the final questionnaire were refusal to continue (*n* = 74) and loss to follow-up (*n* = 21). In the final questionnaire, participants were asked whether they were more bothered, less bothered or just as bothered by the HDM allergy at the end of the survey as at the start (Fig. 7). When considering the overall study population, there was strong trend towards improvement: the proportion of patients with improved overall status (50%) far outweighed the proportion of patients with worsened overall status (7%). There were slight country-to-country differences, with the greatest proportion of patients with improved overall status (63%) observed in France. The time trends for the 85 HDM-only patients did not differ markedly from the overall results (data not shown). The mean scores in Italy, France and Spain were, respectively, 6.9, 7.0 and 7.3. Overall, the level of disease control appeared to increase slightly over the study period.

## Discussion

Taken as a whole, the results of the present observational study emphasize that HDM allergy is heterogeneous (the majority of people with HDM allergy were rarely bothered or not bothered for much of the time) but can be extremely burdensome (i.e. bothersome symptoms every day of the year) for a non-insignificant minority of sufferers (14% in Italy, 46% in France and 37% in Spain). The present results notably agreed with recent literature data on HDM allergy in the countries studied here [7–9]. However, the relationships between allergen sensitization (and especially HDM sensitization) and symptoms are complex and contrasting. For example, a recent study of time patterns of component-specific serum IgE levels from infancy to adolescence and the patterns’ relationship with allergic diseases found that sensitization to HDMs at the age of 16 was associated with an increased risk of asthma and wheeze at the same age [17]. However, this was not the case for sensitization to HDMs at the age of 5 and clinical disease at 16. Similarly, sensitization to HDMs at the age of 5 was not predictive of rhinitis at the age of 16 [17].

Interestingly, we found that professional activities were more affected than social activities; this might be due to the fact that the most frequently impacted disease burden variables in the present study population were sleep, daytime tiredness, and irritability. This confirms the results of a study of 907 adults and 843 children suffering from HDM allergy in France; 50.3% of the adults and 37.3% of the children reported poor-quality sleep [18]. The researchers emphasized the significant impact on patients with AR induced by HDM allergy—particularly for persistent, severe AR [18].. In a different work-related setting, a study in the USA found that AR was the greatest contributor—more than asthma, stress, migraine, depression, respiratory infections and hypertension—to loss of productivity in a group of 826 employees at 47 locations [19]. This contribution was mainly due to greater sick leave in AR sufferers.

In contrast, a French study of adolescents and adults consulting specialists for AR (albeit induced by grass pollen and not HDMs) in France found that work and classroom impairments (as assessed with the Work Productivity and Activity Impairment Questionnaire plus Classroom Impairment Questions: Allergy Specific) were only weakly correlated with AR symptom scores [20].

This self-reported, observational, Internet- and telephone-based survey had several limitations by virtue of its design, as described previously [12]. Firstly, recall bias may have been associated with the final questionnaire, in which participants had to recall events and opinions from at least 13 months earlier. Secondly, women and young adults were probably over-represented in the study population, as these groups are more likely to be Internet users [21]. Thirdly, the participants’ self-reported data on symptoms and QoL were recorded with custom questionnaires rather than psychometrically validated tools (such as the Rhinoconjunctivitis Quality of Life Questionnaire) [22, 23]. Fourthly, the fortnightly telephone interviews suffered from a variable completion rate (which averaged 75% but ranged from 29 to 97%, depending on the country and the period), with the lowest values during the summer holiday period and at the end of the year. Fifthly, indoor HDM allergen levels and outdoor pollen levels were not measured (for cost reasons), so time trends in these disease-causing variables could not be compared with disease burden variables. Sixthly, the elevated incidence of symptom exacerbations during the autumn period might be partly related to a confounding effect of viral respiratory tract infections in people with respiratory allergies [24]. Seventhly, the ARCT was administered in the fortnightly questionnaire but not in the post-inclusion or final questionnaires, where a self-reported 0-to-10 numerical scale was used to rate the degree of disease control. This difference may have led to the slight mismatch between the obligatory self-reported poor disease control at inclusion (an inclusion criterion) and the levels measured with the ARCT. Eighthly, the participants’ use of proton pump inhibitors was not recorded separately, even though these medications can influence allergies [25]. Ninthly, a majority of the patients were sensitized to aeroallergens other than HDMs, and so the results for the study population as a whole could have been biased by related changes in exposure. However, the study results for population as a whole were always similar to the results for the “HDM-only” group. Lastly, and even though the study was an observational survey of ‘real-life’ clinical practice, it may be that the intense study monitoring (with fortnightly questionnaires) itself induced a decrease in the symptoms experienced (i.e. a type of placebo effect), as has been seen in clinical trials in the field of AR [26].

Conversely, our study had some notable strengths: its international, multicentre, longitudinal design, the relatively large number of participants (at least when compared with the 37 participants in a previous study of this topic [11]), the detailed post-inclusion and final questionnaires and the high frequency of follow-up interviews.

## Conclusion

In a 13-month, international, multicentre survey, we found that a relevant minority of patients with a self-reported history of moderate-to-severe, poorly controlled AR and asthma induced by HDMs and potentially by other aeroallergens regularly reported a severe or very severe disease burden in terms of tiredness, impaired sleep and perturbed professional activities (including time off work). Although the level of impairment was heterogeneous, there were clear peaks in the disease burden in autumn and late spring. The level of disease control varied over the course of the study but time trends were not apparent. Encouragingly, there was an overall time trend towards a reduction in the disease burden as treatment went on: the proportion of patients with improved overall status was far greater than the proportion of patients with worsened overall status. In future work, we intend to analyse the possible relationships between worsened status on one hand and demographic, disease-related and treatment-related factors on the other.

## Supplementary information

**Additional file 1: Table S1.** Characteristics of the survey population, according to the post-inclusion questionnaire [12].

**Additional file 2: Figure S1.**Fortnightly telephone interviews of ‘HDM-only’ participants: the impact of the HDM allergy on six types of activity from late May 2012 to December 2012, shown against the symptom intensity. The size of the bubble is proportional (albeit not exactly) to the proportion of participants reporting an impact.

**Additional file 3: Figure S2.**Fortnightly telephone interviews of ‘HDM-only’ participants: the impact of the HDM allergy on six types of activity from Jan 2013 to late July 2013, shown against the symptom intensity. The size of the bubble is proportional (albeit not exactly) to the proportion of participants reporting an impact.

## Data Availability

The proprietary datasets generated and/or analysed during the current study are not publicly available but are available from the corresponding author on reasonable request.

## Abbreviations

AR: Allergic rhinitis
ARCT: Allergic rhinitis control test
ENT: Ear, nose and throat
GP: General practitioner
HDM: House dust mite
QoL: Quality of life
