# Supplementary material for: The disease burden in patients with respiratory allergies induced by house dust mites: a year-long observational survey in three European countries
Source: Clin Transl Allergy. 2020 Jul 1;10:27. doi: 10.1186/s13601-020-00331-0 (PMC7328274; doi:10.1186/s13601-020-00331-0)
Supplement: Supplementary file 1 — Additional file 1: Table S1. Characteristics of the survey population, according to the post-inclusion questionnaire [12]. [file 13601_2020_331_MOESM1_ESM.docx]

**Additional Table S1.** Characteristics of the survey population, according to the post-inclusion questionnaire [12].

|  | Italy | France | Spain |
| --- | --- | --- | --- |
| Number of participants (*n* = males/females), % female | 114 (36/78), 68% | 92 (27/65), 72% | 107 (41/66), 61% |
| Age (years) mean, median [range] | 37.5, 36 [18–63] | 35.8, 36 [18–62] | 38.2, 37 [18–68] |
| Time since first symptoms (years) mean, median [range] | 15.0, 16 [1–45] | 17.8, 18 [1–51] | 17.3, 13 [1–40] |
| Time interval between first symptoms and consultation with a specialist (months) mean, median [range] | 18.9, 4 [0.25–588] | 28.4, 6 [0.25–360] | 20.3, 11 [0.25–156] |
| Other self-reported allergies (% of patients):  grass pollen  *Parietaria* pollen  cat dander  dog dander  olive pollen  birch pollen  moulds  cypress pollen  none (i.e. HDMs only) | 79  57  49  31  27  27  24  21  39 | 67  37  51  26  41  44  29  48  21 | 67  9  41  29  35  7  35  9  21 |
| Proportion of patients having consulted the following physicians, % (mean number of visits per year)  GP  allergologist  ENT specialist  dermatologist  pulmonologist  paediatrician  other | 92 (3.4)  87 (2.0)  27 (2.3)  24 (1.6)  22 (1.5)  14 (6.8)  6 (4.0) | 91 (3.0)  83 (1.7)  27 (2.3)  20 (1.7)  32 (1.6)  9 (1.0)  2 (1.5) | 70 (3.9)  70 (1.4)  15 (1.7)  8 (2.6)  8 (6.2)  7 (n.a.)  4 (1.0) |
| Proportion of patients having consulted the following combinations of physicians, %  GP only  allergologist only  GP + allergologist  GP + another specialist  GP + two specialists  GP + three or more specialists | 17  18  33  7  11  4 | 9  4  25  7  29  22 | 5  3  31  5  26  28 |
| Prevalence of co-morbidities, % of patients  sinusitis  otitis  conjunctivitis  headache | 36  13  44  69 | 53  18  43  62 | 26  9  33  69 |
| Time having used symptomatic medications, % of patients  less than 2 years  2–5 years  6–10 years  11–20 years  more than 20 years | 7  35  22  21  16 | 3  28  39  23  7 | 3  21  30  27  15 |
| Degree of disease control, % of patients  Totally controlled  Well controlled  Moderately controlled  Poorly controlled  Not controlled at all | 11  43  42  4  0 | 4  53  34  7  2 | 10  48  33  7  3 |
| Proportion of patients suffering from symptoms for more than 4 days in a week, % | 70 | 58 | 54 |
| Proportion of patients taking medications, %  antihistamines  nasal corticoids  inhaled corticoids  bronchodilators  inhaled corticoids + bronchodilator  oral or topical corticoids  eye drops  leukotriene receptor antagonists | 38  7  3  7  5  12  4  2 | 40  12  3  12  7  3  6  4 | 34  10  9  15  5  4  4  3 |

HDM: house dust mite; GP: general practitioner; ENT: ear, nose and throat; n.a. not available
