# Supplementary figures and images for: The disease burden in patients with respiratory allergies induced by house dust mites: a year-long observational survey in three European countries
Source: Clin Transl Allergy. 2020 Jul 1;10:27. doi: 10.1186/s13601-020-00331-0 (PMC7328274; doi:10.1186/s13601-020-00331-0)

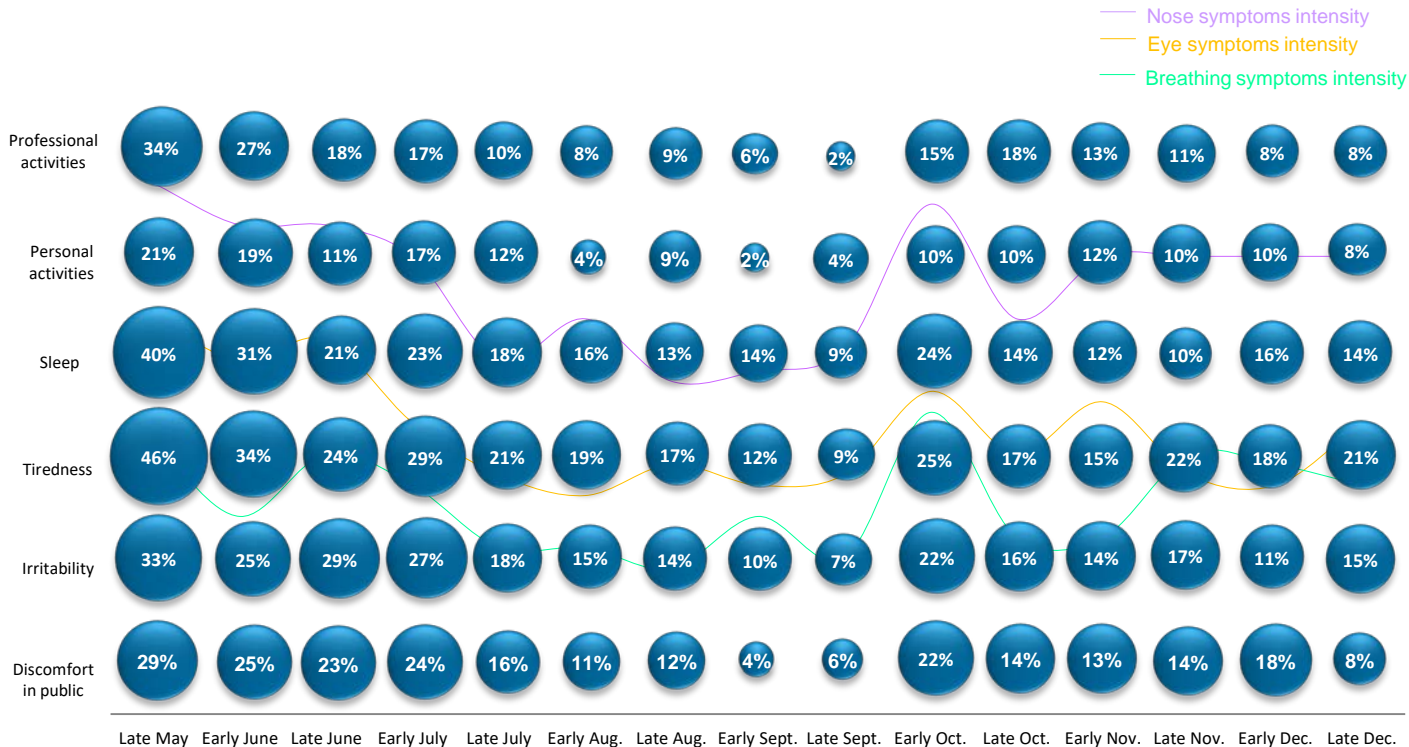

Supplement: Supplementary file 2 — Additional file 2: Figure S1.Fortnightly telephone interviews of ‘HDM-only’ participants: the impact of the HDM allergy on six types of activity from late May 2012 to December 2012, shown against the symptom intensity. The size of the bubble is proportional (albeit not exactly) to the proportion of participants reporting an impact. [file 13601_2020_331_MOESM2_ESM.pdf]

Nose symptoms intensity  
Eye symptoms intensity  
Breathing symptoms intensity

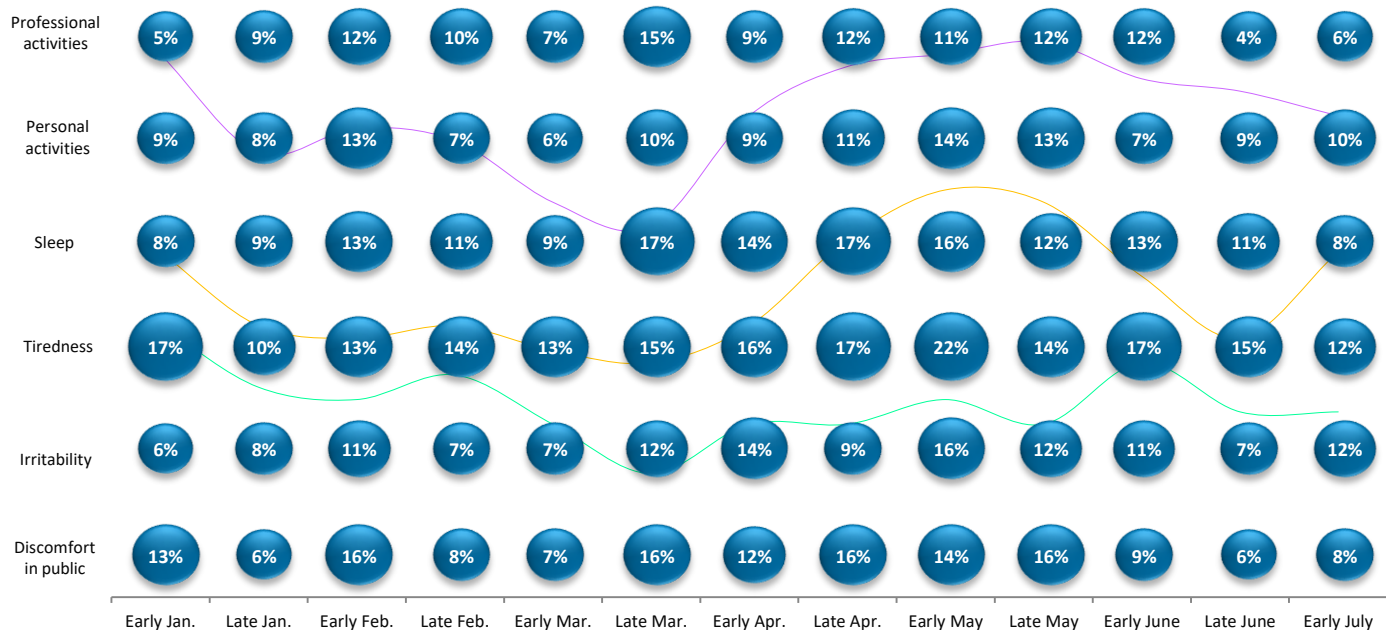

Supplement: Supplementary file 3 — Additional file 3: Figure S2.Fortnightly telephone interviews of ‘HDM-only’ participants: the impact of the HDM allergy on six types of activity from Jan 2013 to late July 2013, shown against the symptom intensity. The size of the bubble is proportional (albeit not exactly) to the proportion of participants reporting an impact. [file 13601_2020_331_MOESM3_ESM.pdf]
